# Enhancing mental health literacy and care through community-driven solutions in rural Bangladesh

**DOI:** 10.3389/fgwh.2024.1478817

**Published:** 2024-12-12

**Authors:** Ryan Afreen, Sanjna L. Surya, Tasnim Jara, Ima Islam, Rahana Parvin, S. M. Ferdousuzzaman, Masum Salah Uddin, Nahreen Ahmed

**Affiliations:** ^1^ Icahn School of Medicine at Mount Sinai, New York, NY, United States; ^2^ Brigham and Women’s Hospital, Boston, MA, United States; ^3^ Cambridge University Hospitals NHS Foundation Trust, Cambridge, United Kingdom; ^4^ Royal Papworth Hospital NHS Foundation Trust, Cambridge, United Kingdom; ^5^ MedGlobal, Rolling Meadows, IL, United States; ^6^ Department of Pulmonary, Allergy, and Critical Care, Hospital of the University of Pennsylvania, Philadelphia, PA, United States

**Keywords:** community-based participatory research (CBPR), needs assessment, women’s autonomy, stigmatization, mental disorders, tech-hubs

## Abstract

Women in rural Bangladesh encounter significant barriers to seeking mental healthcare, primarily due to stigmatization rooted in a lack of knowledge about mental health. To address this issue, community-based participatory research (CBPR) has been identified as a promising approach. CBPR involves the active collaboration of community members and stakeholders in the research process to tackle pressing community issues. This study examines the application of CBPR to enhance mental health awareness and education among women. The program described in this study leverages local technology centers and community health workers to boost participation in improving access to equitable mental healthcare and increasing health literacy. Implemented across three rural districts in Bangladesh, the program began with a needs assessment survey to evaluate participants' knowledge, experiences, and stigmatization of psychological conditions such as depression, anxiety, psychosis, suicide, and postpartum depression. This survey assessed baseline knowledge, personal experiences, and the perceived impact of mental wellness as a community issue. Additionally, five focus group discussions were conducted at local tech hubs with trained community health workers to explore participants' perceptions and attitudes toward mental health. These discussions highlighted the role of medical misinformation, the shortage of professionals, and other factors contributing to mental health stigmatization. The study's second phase would focus on developing digital medical content for screening at tech hubs, followed by live Q&A sessions with Bangladeshi health experts. This initiative aims to familiarize women with user-friendly telehealth services, fostering healthcare literacy and improving well-being in rural Bangladesh.

## Introduction

The stigma surrounding mental health has long been a significant challenge in Bangladesh. From widespread beliefs in the nonexistence of psychological disorders to attributing symptoms to paranormal activities, mental health is met with substantial scrutiny. It is estimated that 15%–35% of the population in Bangladesh experiences behavioral health challenges (1). However, studies suggest a lack of a comprehensive review of the nation's current mental health status (2). Resource-poor countries, specifically, suffer from a lack of mental health awareness, education, and specialists (3). Globally, the stigmatization of mental health is recognized as a large contributing factor responsible for the avoidance of mental health care (4–6). Stigma is viewed as a disgrace, social disapproval, or discrediting of individuals with psychological disorders (7). This includes labeling, stereotyping, prejudice, rejection, social isolation, status loss, ignorance, low self-esteem, low self-efficacy, discrimination, and marginalization of individuals with mental health challenges (8, 9).

A national mental health survey conducted before the COVID-19 pandemic found that only 11.6% of women in Bangladesh accessed mental healthcare (10). Health-seeking behavior is significantly less among women living in rural areas (11). Many women in the postpartum period have several risk factors for progressive mental health disorders including a prior history of mental health disorders, poverty, insufficient nutrition, physical violence, domestic abuse, and stress disorders (12, 13). These risk factors, compounded by stigmatization, discourage women in rural Bangladesh from seeking mental health care. Widespread stigmatization is often attributed to a lack of knowledge and superstitious beliefs about the causes of psychological conditions (14, 15).

The role of women's autonomy in improving health outcomes in Bangladesh has been overlooked (16–18). Community-based participatory research (CBPR) offers an empowering and effective way of collaborating with target populations to enhance health outcomes within their communities (19). Through CBPR, a target population can systematically uncover the unaddressed needs within their communities through active participation and collaboration, influencing every aspect of the process, from research to interventions (19). One of the core CBPR principles is a needs assessment, which highlights gaps, needs, and assets within the community to develop an action plan (20).

The objective of this IRB-approved study is to utilize the CBPR framework to identify and address the lack of education on psychological conditions and mental wellness among women in rural Bangladesh by leveraging local infrastructure. We hypothesize that by using local resources to conduct a needs assessment, we will be able to:

1. Empower women to combat stigmatization within their communities,
2. Improve health-seeking behaviors with the help of local, sustainable resources that are convenient to access.
3. Improve mental health literacy

## Geographical context

Bangladesh, a lower-middle-income country in South Asia, is the 8th most populous country in the world (21). The country faces significant challenges in hospital resource availability for both communicable and non-communicable diseases, with only 4 hospital beds per 10,000 people (22, 23). Specifically, within the mental health sector, only 8% of the total hospital beds are designated for mental health patients, and there are no beds available for mentally ill patients in forensic units across the country (23). Bangladesh has only one mental hospital, established in 1957 (24). Furthermore, there are only 0.49 trained and skilled mental health professionals per 100,000 population (23). With minimal economic investment and medical training in mental health services, mental healthcare in Bangladesh is hindered by stigmatization (25).

The crippling effect of the stigma associated with mental illness impacts all domains of life (6, 9, 26). Existing literature reveals that stigma diminishes self-esteem (27, 28) and self-efficacy (29, 30), and deprives affected individuals of social benefits, support systems, provider networks, and community resources (26, 28). Socioeconomic disadvantages, combined with the significant burden of household responsibilities, result in women's mental health needs often being overlooked (31). Low mental health literacy further impacts health-seeking behavior among women (32, 33). Women in Bangladesh are more likely to be diagnosed with prevalent psychological challenges but are less likely to seek treatment compared to their male counterparts (34, 35).

Interpersonal relationships are also adversely impacted by mental illness stigma (36–38). Increased behavioral health challenges, decreased coping skills, and reduced compliance with treatment are associated with mental illness stigma (39, 40). The stigma surrounding mental disorders not only affects psychological well-being but also leads to physiological consequences such as obesity, back pain, and sexual health issues, as stress and anxiety stemming from stigma can manifest in various physical health problems (6). Considering its pervasive impact, stigma has been identified as a major social concern for individuals with mental health challenges and their families, even in this age of the rapid emergence of psychological disorders globally (6).

Although women in Bangladesh are more likely to suffer from depression compared to their male counterparts, they are also hesitant to express or share their health concerns (23, 41, 42). Our primary concern regarding our intervention is whether women in rural areas will be willing to participate in the needs assessment, which aims to investigate their knowledge gaps and willingness to learn and express their mental health concerns.

## Why CBPR?

CBPR is centered on the concept of community, offering a vital means of engaging local community members alongside stakeholders to develop solutions for issues within their communities. CBPR combines knowledge with action to foster positive social change and encourages collaborative efforts to create sustainable solutions to reduce health disparities (43, 44). Research shows that CBPR is particularly effective in psychology, enhancing efforts to address mental health disparities in access, treatments, and initiatives for marginalized groups (43, 45). Its adaptable approach makes it suitable for diverse community partnerships, fostering trust, co-learning, and cultural humility (43).

Practitioners of CBPR recognize that individuals are deeply connected with their communities through shared interests and a collective commitment to meeting community needs (43, 46). This is especially relevant in rural areas where women, who are not only geographically connected, also play key roles in agricultural and non-agricultural sectors (47). The CBPR framework allows participants to reflect on how their social identities (e.g., race, ethnicity, gender, education, socioeconomic status) influence their own and their community's engagement in research, thereby helping to dismantle power imbalances (43). By cultivating a community of female health advocates, CBPR effectively addresses the barriers to care by integrating their lived experiences with scientific evidence (43).

CBPR integrates education and social actions to address health disparities and outcomes (48). This approach is crucial in destigmatizing mental healthcare in rural communities in Bangladesh. Implementing health education and fostering a community of mental health advocates can significantly improve overall health outcomes (49–51). Through CBPR, women in the community can share their insights to help formulate culturally sensitive research questions that identify key behavioral health challenges (52). This collaborative approach is essential for understanding the mental healthcare needs within a community and for implementing policies, legislation, and benefits for individuals with psychological disorders (50).

Additionally, CBPR is guided by a multideterminant perspective, recognizing that familial, community, societal, and geopolitical factors significantly influence health status (53). This perspective led to the creation of a diverse team in our program, consisting of physicians, public health experts, social workers, non-governmental organizations (NGOs), and community workers.

## Program implementation

Our program was implemented in three rural districts of Bangladesh: Joypurhat, Pabna, and Naogaon. We partnered with “tech-hubs”, local technological centers founded by the SBK Foundation, to conduct the needs assessment survey. SBK Foundation is a Bangladeshi non-profit organization and the world's first Digital and Shariah-compliant licensed Microfinance Institution (MFI), established to empower women and youth through technological equity (54).

On the ground, the project was implemented in collaboration with a team of tech-hub staff, female social and community health workers, and research professionals from MedGlobal. MedGlobal is an international nonprofit organization that has been providing humanitarian services in Bangladesh since 2017 to improve local health systems and support marginalized communities (55). The research team developed a comprehensive needs assessment survey that covered three main areas: (1) mental health, (2) reproductive health, and (3) preventative and primary care.

The needs assessment evaluated participant demographics, identifying gaps and barriers in education and perceptions of mental health. Topics assessed included knowledge of various psychological disorders and challenges related to postpartum mental health. Participants were asked about their understanding of medical terminologies (e.g., “Which of the following matches your understanding of schizophrenia?”), experiences with mental health disorders, consultation with specialists, availability of specialists in the community, treatment received, and barriers to care. Questions were both multiple-choice and open-ended (Supplementary Material).

The second phase of the study involved focus group discussions using semi-structured interview questions. These discussions allowed women to address their health concerns, share personal narratives, and seek advice on organizing community efforts for tangible mental wellness services. The focus groups also explored how misinformation and lack of community and family support contributed to the stigmatization of psychological disorders, as well as the reluctance to address their health concerns. The discussions were aimed at highlighting mental health issues prevalent in the community, exacerbated by a lack of awareness, resources, and stigma.

During the focus group discussions, the women quickly took control, guiding the conversations and asking questions beyond the semi-structured interview format. This pivotal moment inspired everyone in the room—no one hesitated to share their individual mental health concerns and experiences with services or the lack thereof. Although the sample size of the intervention is small, it is a valuable study that serves as a stepping stone towards demystifying mental health education and gradually eliminating stigma.

## Discussion

While the stigma around mental health persists in rural Bangladesh, our pilot initiative was created to embrace health literacy to build an informed and healthier community. The focus group discussions centered around themes that continue to influence stigmatization: (1) the stigma around seeking mental health care, (2) medical misinformation spread through social media, (3) the lack of affordable and accessible healthcare providers (i.e., lack of psychiatric care centers), and (4) the lack of telemedicine services. The discussion encouraged the women to elaborate on their experiences of encountering these barriers to care and how they shaped their health-seeking behaviors (Table 1).

**Table 1:** Barriers to mental healthcare that influence health-seeking behavior.

Barriers	Description
Stigmatization	Women were isolated from community or experienced discrimination when they sought mental healthcare services
Medical misinformation	Prevalence of medical misinformation through social media exacerbated misunderstandings and stigmatization of mental health
Lack of providers	There is a lack of mental healthcare clinics and providers in rural areas of Bangladesh
Lack of telemedicine	Limited technological infrastructure and lack of mental healthcare providers result in lack of telemedicine services in the rural areas

In the next phase of the initiative, a new partner was incorporated, called Shohay, an organization with an e-platform that provides reliable medical information (diagnosis, symptom management, and treatment options) to Bengali-speaking populations worldwide (56). In collaboration with Shohay, a three-part video series was created, explaining different aspects of psychological disorders, symptom management, and available treatment options. The video series would be screened for cultural and language appropriateness for the target audience.

To assess the effective delivery of medical information and knowledge retention, and determine the overall impact of the study, our pilot initiative was implemented in two steps. First, a survey post-screening was administered to address any questions or concerns from the information provided in the video series. Following that, in partnership with a group of Bengali-speaking physicians and medical experts from Shohay and MedGlobal, virtual Q&A sessions were hosted at the tech hubs. These sessions provided a safe space for the women to ask questions, seek medical advice and guidance, and build their knowledge about psychological disorders and treatment. Each live Q&A session was conducted weekly.

## Conclusion

We believe this initiative would help mitigate the lack of accessible and affordable medical care specialists and the availability of accurate medical information. To evaluate long-term changes in women's health-seeking behavior, we would continue to administer mid-year surveys and host focus group discussions. Our study highlights the effectiveness of CBPR in enhancing mental health awareness and care in rural Bangladesh. By leveraging local resources, our initiative tackled stigma through the promotion of health literacy, facilitated sustainable health-seeking behaviors by encouraging women to actively participate in various aspects of the study, and provided new ideas and insights on barriers to care. Future studies should explore the longitudinal impacts of CBPR on health behaviors in different resource-poor settings and introduce telehealth to address the lack of mental health services in underserved communities.

## Data Availability

The original contributions presented in the study are included in the article/Supplementary Material, further inquiries can be directed to the corresponding author.
