# Supplementary material for: Enhancing mental health literacy and care through community-driven solutions in rural Bangladesh
Source: Front Glob Womens Health. 2024 Dec 12;5:1478817. doi: 10.3389/fgwh.2024.1478817 (PMC11669598; doi:10.3389/fgwh.2024.1478817)
Supplement: Supplementary file 1 [file Table1.docx]

**FOCUS GROUP QUESTIONS**

**Focus Group A: Mental Health**

1. 83% of women stated that mental health disorders are a big problem in their community, with anxiety and depression followed by schizophrenia being the most common diseases. What do you think of these results? Do they surprise you?

2. Most women mentioned that sadness is caused by laziness, anxiety is caused by inability to cope with normal stresses of daily life, and schizophrenia is caused by chemical imbalances in the brain as well as by intentional acts of abnormality. The medical definitions of these disorders are as follows:

a. Depression is defined as persistent feelings of deep sadness resulting in poor motivation and loss of interest, caused by stressful events, impairment of neural circuits, hormonal imbalances, and other biological and social events.

b. Anxiety is defined as intense, excessive, and persistent worry and fear of everyday situations that are caused by stressful life events, chemical imbalances in the brain, or health conditions such as thyroid problems.

c. Schizophrenia is a disorder that affects a person’s ability to think, feel, and behave normally and is caused by genetics, altered brain factors, and environmental factors.

What do you think of these definitions? Do they surprise you?

3. 50% of women mentioned that barriers to seeking care for mental health disorders include taboo and stigma around mental health disorders.

a. What are some reasons these disorders are seen as taboo?

b. What are ways we can decrease stigma around these disorders?

c. Would you be interested in interventions that help spread awareness in your community around mental health? Do you think your community would benefit from such interventions? Why/why not?

d. Would you be interested in group therapy sessions run by doctors or nurses or family members? Do you feel the availability of such sessions would be beneficial to your community? Why or why not?

e. Would you be interested in watching educational videos on mental health diseases and prevention at tech hubs?

4. 65% of women mentioned that they experienced a mental health disorder but chose not to speak with a medical or mental health professional about it.

a. How often have you heard of people in your community seeking mental health treatment?

b. From whom do they seek treatment and what is their training?

c. How far away do they have to travel to seek mental health consultation and treatment?

d. How much do such visits cost?

e. Do you think doctors spend adequate time addressing mental health issues?

f. If you or others in your community do not consult doctors or family members when experiencing depression or anxiety, what do you do instead?

5. Is there a need for more resources in your community for diagnosing and treating people with mental health disorders?

a. If yes, what are the best ways to deliver these resources?

b. Would you prefer to access them through nearby clinics and hospitals, through telehealth services, or both?

6. Is extreme sadness after delivery an important problem in your community? Do you know of people who have experienced this? What is your understanding of the causes of this?

7. Feelings of extreme sadness after pregnancy is called postpartum depression that can be treated. What resources are available for women in your community seeking treatment for postpartum depression?

8. Would you or others in your community be interested in learning more about screening and treatment for postpartum depression?

9. From where do you receive most of your information on mental health disorders such as depression, anxiety, schizophrenia, and postpartum depression? Social media, word of mouth, school/college, other?

10. What are the biggest barriers to women in your community accessing mental health treatments?

a. Is there a stigma against taking medications to treat mental health disorders in your community?

b. Do you think there is a need for doctors and nurses who treat people in your community to be trained on screening and treating mental health disorders?
